# Inflammation and Metabolism in Cancer Cell—Mitochondria Key Player

**DOI:** 10.3389/fonc.2019.00348

**Published:** 2019-05-14

**Authors:** Monica Neagu, Carolina Constantin, Iulia Dana Popescu, Donato Zipeto, George Tzanakakis, Dragana Nikitovic, Concettina Fenga, Constantine A. Stratakis, Demetrios A. Spandidos, Aristidis M. Tsatsakis

**Affiliations:** ^1^Immunology Laboratory, Victor Babes National Institute of Pathology, Bucharest, Romania; ^2^Doctoral School, Biology Faculty, University of Bucharest, Bucharest, Romania; ^3^Pathology Department, Colentina Clinical Hospital, Bucharest, Romania; ^4^Department of Medicine, University of Pittsburgh, Pittsburgh, PA, United States; ^5^Department Neuroscience, Biomedicine and Movement Science, School of Medicine, University of Verona, Verona, Italy; ^6^Laboratory of Anatomy-Histology-Embryology, Medical School, University of Crete, Heraklion, Greece; ^7^Biomedical, Odontoiatric, Morphological and Functional Images Department, Occupational Medicine Section, University of Messina, Messina, Italy; ^8^Section on Genetics & Endocrinology (SEGEN), Eunice Kennedy Shriver National Institute of Child Health & Human Development (NICHD), NIH, Bethesda, MD, United States; ^9^Laboratory of Clinical Virology, Medical School, University of Crete, Heraklion, Greece; ^10^Department of Forensic Sciences and Toxicology, University of Crete, Heraklion, Greece

**Keywords:** metabolic pathways, inflammation, cancer cell, tumorigenesis, therapy targets

## Abstract

Cancer metabolism is an essential aspect of tumorigenesis, as cancer cells have increased energy requirements in comparison to normal cells. Thus, an enhanced metabolism is needed in order to accommodate tumor cells' accelerated biological functions, including increased proliferation, vigorous migration during metastasis, and adaptation to different tissues from the primary invasion site. In this context, the assessment of tumor cell metabolic pathways generates crucial data pertaining to the mechanisms through which tumor cells survive and grow in a milieu of host defense mechanisms. Indeed, various studies have demonstrated that the metabolic signature of tumors is heterogeneous. Furthermore, these metabolic changes induce the exacerbated production of several molecules, which result in alterations that aid an inflammatory milieu. The therapeutic armentarium for oncology should thus include metabolic and inflammation regulators. Our expanding knowledge of the metabolic behavior of tumor cells, whether from solid tumors or hematologic malignancies, may provide the basis for the development of tailor-made cancer therapies.

## Introduction

Overall, a normal cell and a tumor cell have very different metabolic status, as one of the main characteristics of the tumor cell is an altered cell metabolism. Indeed, the dynamic metabolism of the tumor cell with increased metabolic fluxes and nutritional needs is required in order to support an accelerated cell proliferation and to enhance other biological functions, including migration, cell response to hypoxia due to accelerated proliferation and adaptation to different tissue environments during the metastatic process.An altered tumor cell metabolism induces the exacerbated production of lactate, nitric oxide (NO), reactive oxygen species (ROS) and arachidonic acid metabolism products, such as prostaglandins that simultaneously contribute to the inflammatory milieu (1). These changes in the local inflammatory status may actually facilitate the development of a tumor-permissive environment. Deregulated metabolism leads to the expression of myriad dysfunctional proteins that contribute to pro-tumorigenic processes. Therefore, altered tumor cell metabolism supports characteristics gained by the tumor cell during the complex process of tumorigenesis: a deregulated cell cycle with enhanced anti-apoptotic characteristics, deregulated cell death pathways, increased migratory potential, and a high adaptability to the tissue microenvironment (2).

Metabolic heterogeneity represents a hallmark in various types of human cancer, where glucose metabolism is the main deregulated metabolic pathway, leading to protein and gene deregulations and hence, to tumorigenesis (3). Within the cell, mitochondria is involved in tumorigenesis through various pathways: ROS levels, DNA mutations and, hence, genomic instability, autophagy control, resistance to cell death stimuli, metabolic alterations as decreased oxidative phosphorylation (OXPHOS) and anabolic pathways induction. Various intracellular signaling events, such as kinase signaling that modulate transcription factors and can further induce epigenetic changes (4).

## Tumor cell metabolism is sustained by mitochondrial biochemical pathways

One of the main hallmarks of tumorigenesis is an altered cell metabolism, where the dynamic metabolism of the tumor cell with increased metabolic fluxes and nutritional needs is required in order to support an accelerated cell proliferation, as well as to enhance other tumor cell biological functions (5).

Indeed, specific metabolic reprogramming facilitates tumor development and all these deregulations can become therapeutic targets. Some therapeutical strategies directed to cancer cell metabolic disturbances have been found to be highly effective, and offer grounds for further development (5).

The main metabolic alterations of the tumor cells include an increased aerobic glycolysis (6), pH deregulation (7), lipid metabolism dysregulation (8, 9), increased generation of ROS (10), as well as disturbances of enzyme activities (11–14). These traits lead to apoptotic pathways alterations, genomic and transcriptomic modification, finally inducing pro-tumorigenic features (15). Moreover, several studies have proposed a link between the altered metabolism that favors tumor cell survival in a “hostile” milieu, such as an ischemic and/or acidic microenvironment, an evasion from the attack of the immune system elements and cancer stem cell resistance (16).

As summarized by Chen et al. the mitochondria exhibit an array of metabolic deregulations in cancer cells, deregulations that are potential therapeutic targets and display selectivity in a cancer type-dependent manner (17). Alterations in intracellular signaling pathways would induce profound mitochondrial changes, followed by reorganization of other cellular compartments. The intracellular pathways deregulation is caused by the activation of oncogenes like Ras or Myc, by the induction of transcription factors such as HIF1α, as well as by the inactivation of tumor suppressor genes like p53. All these abnormal molecular imprints induce enhanced glucose intake and inhibition of oxidative phosphorylation system (OXPHOS), not related to oxygen availability, summing up the aerobic glycolysis or Warburg effect. As a direct consequence, the activation of pentose phosphate pathway or amino acid biosynthetic pathways generates high levels of anabolic intermediates. In the meantime, ROS scavenging systems are enhanced and protect tumor cells from oxidative-type of actions. The extracellular microenvironment becomes more acid and, thus, it increases the activity of several pro-invasive factors (18, 19). The tumor cell has increased anabolic needs now; therefore, there is an increased lipid biosynthesis (20) using glutamine for fueling tricarboxylic acid (TCA) cycle. The overall consequence is that the tumor cell proliferates even when hypoxia is installed due to rapidly expanding tumor mass, low blood/nutrients addition, and oscillations of redox conditions (21, 22). This complex metabolic adaptation is governed by mitochondria that harbor the effector systems for the bio-energetic platform of tumor cells. This organelle is the active inducer of metabolic rewiring of the tumor cell, such as mutations in genes that code respiratory chain proteins which are associated with various tumor types (4).

Another metabolic deregulation in cancer cells is an alteration of the lipid metabolism that also supports increased cell growth. Fatty acids (FA) are needed by cancer cells for various functions: signaling molecules, membrane building molecules and bioenergetic substrates. It has been recently demonstrated that the expression of adipose triglyceride lipase (ATGL) which catalyzes triacylglycerols (TAGs) hydrolysis is down-regulated in major solid cancers, such as lung, muscle and pancreatic cancers (23–25). It seems that the ATGL involvement in cancer cell metabolism is linked to the peroxisome proliferator-activated receptor-α (PPAR-α) signaling and to the pathways involved in inflammation, redox homoeostasis and autophagy (26–28). As recently discussed by Vegliante et al. these cellular processes are strongly implicated in tumorigenesis initiation and metastasis (29). Importantly, ATGL down-regulation can also be involved in the switch from a mitochondrial metabolism to a glycolytic type; this metabolic switch is characteristic to the majority of tumors (30).

Lipid metabolism can affect mitochondrial *cristae* morphology and consequently, the mitochondrial activity. Indeed, any deregulation of the lipid metabolism will modulate mitochondrial function due to the lipid role in the maintaining of the bio-membranes integrity (31, 32). As the mitochondria are intracellular organelles that play a crucial role in cell metabolism by producing ATP through OXPHOS, a decrease in OXPHOS expression due to mitochondrial lipid modulation will result in OXPHOS activation and an increased alternative energy requirement (33). Importantly, in the mitochondria, cardiolipin accounts for a major 20% of the total lipid mitochondrial composition. In tumor cells, an abnormal cardiolipin level has been identified (34). As OXPHOS processes generate large quantities of protons that induce important pH alterations, under normal conditions, cardiolipin traps protons within the mitochondrial membrane, minimizing the pH changes (35). The protective mechanism is overridden in tumor cells, leading to mitochondrial activity dysfunction (36). Indeed, as suggested by Kiebiesh et al. in tumor cells, lipid and electron transport dysfunctionalities of the mitochondria are hallmarks of metabolic deregulations (37). Of note, as normal and tumor cells have very different energy metabolism rates, which can be affected by *in vitro* conditions, caution is needed when interpreting metabolic data of malignant vs. non-malignant cells under *in vitro*/*in vivo* conditions (31).

Enzymes that control deregulated metabolic pathways and proton cycles are important therapeutic targets in cancer. Thus, upregulated enzymes involved in cancer cell bioenergetics and biosynthesis can be shut down by specific inhibitors. In a recent study by Yadav et al. it was reported that 3-bromopyruvate [3-BP] can inhibit several metabolic enzymes (38). Specifically, an *in silico* approach that was used indicated that 3-BP can target glycolysis enzymes and enzymes involved in the TCA cycle. Furthermore, derivatives of 3-BP, dibromopyruvate (DBPA), and propionic acid (PA) were shown to have an increased binding affinity to metabolic enzymes. This approach demonstrates the feasibility of utilizing metabolic enzyme inhibitors for anti-cancer therapy (38).

As glutamine metabolism often depends on mitochondrial glutaminase (GLS) activity, GLS has become a target molecule for developing new potent inhibitors for GLS and, as recently reported, CB-839 chemical compound has entered clinical trials for advanced solid tumors and hematological malignancies (39).

The enzyme 6-phosphofructo-2-kinase/fructose-2,6-bisphosphatase 4 (PFKFB4) that controls glycolysis (40) was shown to regulate transcriptional reprogramming through the oncogenic steroid receptor coactivator-3 (SRC-3) (41). Since PFKFB4 is an enzyme that stimulates glycolysis, PFKFB4-mediated SRC-3 activation triggers the pentose phosphate pathway and activates purine synthesis by up-regulating transketolase (41).

### Redox Status

Another metabolic trait of tumor cells is the enhanced ROS generation. As already stated, mitochondria is one of this the main intra-cellular ROS generation organelle and mitochondrial ROS generation is associated with the respiratory chain complexes (42).

As the oxidative metabolism is enhanced in cancer cells, high quantities of ROS are produced by the mitochondrial electron transport chain (ETC), that further activate signaling pathways which are in the vicinity of mitochondrion system promoting cancer cell proliferation (43). However, if the ROS will accumulate in high quantities, cells will undergo apoptosis (44); consequently, tumor cells will generate high quantities of NADPH in the mitochondria and in the cytosol, in order to limit the accumulation of ROS (45). Therefore, both glucose-dependent metabolism and mitochondrial metabolism are highly involved in tumor cell proliferation.

In the redox tumoral context, mitochondrial DNA (mtDNA) and mitochondrial proteins have been shown to be extremely ROS-sensitive due to their vicinity to the respiratory chain (RC). Aiding tumorigenesis, the mitochondrial ROS leads to the accumulation of oncogenic DNA abnormalities and further activation of potentially oncogenic signaling pathways (46).

### Energy Metabolism

The major biochemical task of the mitochondria is the production of ATP, accompanied by the metabolites used for the bioenergetic and biosynthetic necessities of the cell; this organelle serves both as catabolic and anabolic metabolism (47).

The majority of ATP in tumor cells is produced by the mitochondria (48) and targeting this energy metabolic loop can be a good therapy option. As the cells from the center of solid tumors survive in a nutrient-poor milieu with reduced glucose and oxygen (49), if a drug is targeted to block mitochondrial ATP, this will lead to apoptosis. Another option is for the tumors that are highly dependent on oxidative phosphorylation for ATP, such as cutaneous melanoma (50). In this case, tumor cells targeted with drugs that hinder mitochondrial ATP production will enter apoptosis because cells will not be able to have a glycolytic compensation (51).

Another type of metabolic-driven therapy is sustained by the inhibitors of the PI3K intracellular signaling pathway, which synergize with therapies that diminish glycolysis (52). In these therapeutic scenarios, compared to normal cells, tumor cells would selectively intake the inhibitors of mitochondrial ATP production (47). In this area, a common anti-diabetic drug, metformin, which can inhibit complex 1 of ETC, has been tested. An extended analysis of several studies has shown that, indeed, patients with pancreatic cancer that were treated with metformin had a prolonged survival (53). Nevertheless, unfortunately, this important clinical discovery could not be duplicated in patients with advanced pancreatic cancer (54, 55), giving research new avenues to search for improved combined therapies (56).

## Inflammation and metabolic deregulation

Tumor cells are obliged to alter their metabolic status in order to adapt to increased nutritional needs and decreased oxygen supplies in the tissue microenvironment, while sustaining their high proliferation rate. These metabolic changes contribute, on the one hand, to the inflammatory milieu (56–58). On the other hand, the modulation of the tumor microenvironment affects various metabolic processes through the regulation of hormone bioavailability (59).

For immune cells, inflammatory immune phenotypes are associated with increased glycolysis (60), while mitochondrial oxidative networks are associated with immune memory generation, but also immune-suppressive phenotypes (61). As previously stated, cancer cells have a deregulated metabolic system and therapies that target glycolysis, pyruvate oxidation, and glutamine metabolism (e.g., 2-deoxyglucose, DCA, or DON) would also protect from a chronic inflammatory status (61). In terms of targeting metabolic traits in immune cells and in cancer cell, there are some differences. This divergence resides in the metabolic difference between immune cell and tumor cell. If a low metabolic inhibition in immune cells will immediately lead to an inflammation abrogation, in cancer cell the deregulated metabolic pathways must be heavily inhibited so that the cells undergo apoptosis. Cancer cells and immune infiltrating cells reside in a specific microenvironment that hosts inter-cellular signaling molecules influencing the metabolic pattern of the cells (62).

There is a series of inflammatory-mediated cells infiltrating the tumors that aid the metabolic deregulations of the cancer cell. One of the most studied inflammatory infiltrating cells is the tumor-associated macrophage (TAM) (63). TAMs reprogram their metabolism with activation of many pathways, such as glycolysis, FAS and altered nitrogen cycle. Altering their metabolism induces an increased production of cytokines and angiogenic factors, aiding the pro-tumorigenic inflammatory pattern of the tumor, thus favoring progression and metastasis (3). A recent study documented that TAMs are “educated” by the tumor cells in order to perform the delivery of essential elements, e.g., iron (64). During the initiation processes of tumorigenesis and subsequent tumor cell growth and metastasis, an adequate iron supply is obligatory to sustain the accelerated metabolism of the tumor cell (65), as TAMs have highly efficient mechanisms of sequestering, transporting and storing iron by utilizing lipocalin-2 and siderophores (64).

Another cell that is found to infiltrate tumor tissue is the T cell comprising several populations. T lymphocytes are subjected to various metabolic regulations during cell activation and further when they develop their effector functions. Thus, it has recently been shown that FAS regulates a newly discovered T-helper sub-population, Th17 cells (66). The inhibition of early FAS can further inhibit Th17 and promote the appearance of regulatory suppressive Tregs cells, a cell population that favors tumorigenesis. This mechanism was discovered by targeting acetyl-CoA carboxylase (ACC1) (67). Indeed, recent studies have shown that fatty acid synthase (FASN) that is downstream of ACC, is a critical metabolic regulator in the appearance of inflammatory Th17 lymphocytes. Therefore, by inhibiting FASN function, studies have shown that IFN-γ production by Th1 and Th1-like Th17 cells is increased (67).

The mitochondrion is also at the center of pro-inflammatory signaling and likewise, the pro-inflammatory milieu can modify mitochondrial physiology (68). DAMPs that are formed upon mitochondria damage contribute to inflammasome formation and caspase-1 activation (68). Various metabolic inducers, ATP and ROS being just few of these, trigger inflammasome activation. Importantly, metabolic-related molecules, such as ATP, induce the assembly of the inflammasome and the initiation of IL-1beta generation, one of the key mediators of inflammation (68) highlighting the interaction of inflammation and metabolism in tumor tissue (69).

As inflammation is directly associated with tumorigenesis (70), anti-tumoral processes that involve the elements of the immune system should be also acknowledged. Indeed, in response to therapy, cancer cells release immune-enhancing danger signals that facilitates the host tissue anti-tumoral response. A specific group of mitocans, including vitamin E analogs, has been defined; these are compounds that induce ROS production in the mitochondria and contribute to the immune-enhancing danger signals (71). Through this mechanism, an array of events is triggered by the mitochondria that ultimately activates the inflammasome and induces an anti-tumor response (71).

Silent mating type information regulation 2 homolog (SIRT1) and Hypoxia Inducible Factor 1 alpha (HIF1α) are two key *metabolic sensors* that are highly involved in the immune responses. Indeed, HIF1α constitutes the target of SIRT1 in generating immune-mediated responses. It has been recently discussed that these metabolic sensors can be involved in the two main types of immune response (72). Thus, in innate immunity, SIRT1 regulates the glycolytic activity of myeloid-derived suppressor cells and influences their functional differentiation, while inducing an increased NAD+ level in monocytes (73). SIRT1-HIF1α tandem links innate and adaptive immunity with SIRT1, which activates inflammatory T cell subsets through NAD+. HIF1α stimulates glycolysis-associated genes and adjusts the levels of ATP and ROS (74). When these metabolic sensors induce deregulations of T-helper functions, various immune-associated diseases arise; cancer is one of them (72).

At the organism's level, cachexia is one of the clearest examples linking inflammation and metabolism in cancer. This metabolic syndrome is present in 80% of cancer patients. The syndrome is characterized by massive weight loss with marked muscle wasting (75, 76). Complex pathways regulate this syndrome, including molecular and biochemical deregulation inflammation-associated mechanisms. The communication between the tumor and the skeletal muscle is still under investigation; however, it has recently been shown that the inflammatory signals which activate an increased catabolism of the muscle are mediated by the exosomes transporting muscle-specific miRNAs (myomiRs) (77). Indeed, myomiRs *per se* modulate inflammatory pathways, and participate in metastasis initiation through the regulation of protein synthesis and degradation in skeletal muscle (77).

The tumor inflammatory local status regulates immune cells toward pro-tumorigenic properties and allows therapeutic modulation (78). Hence, novel therapeutic targets that will aim metabolic important nodes would decrease the inflammatory status and reprogram immune cells for an anti-tumoral response (79). It is therefore suggested that an enhancement of the anti-tumoral response of the immune system would overcome therapy resistance (1).

## Dysfunctional proteins/deregulated transcriptomics affect tumor cell metabolism

Deregulated cell metabolism results in a myriad of dysfunctional proteins that ultimately facilitate pro-tumorigenic processes. Hence, aiming at specific metabolic nodes can lead to novel approaches in cancer therapy. For example, Verlande et al. examined the effect of metabolic stress on ERK pathway in melanoma cells characterized by NRAS or BRAF oncogenes mutations (80). This approach showed that the two genomic subtypes react differently when a higher level of metabolic stress is induced. Metabolic stress in NRAS-mutant cells, activates the ERK pathway while in BRAF V600E-mutant cells the respective pathway is down regulated. Therefore, this recent study underlines that the oncogene activation is affected by the metabolic particularities of tumor cells (80). Likewise, KRAS mutations detected in various human cancers, e.g., pancreatic cancer, colorectal cancer, and non-small cell lung cancer were shown to play a role in tumor cell aerobic glycolysis, as recently discussed (81). Indeed, tumors with KRAS mutations have an enhanced nutrient uptake, increased glycolysis and glutaminolysis, and an increased synthesis of fatty acids and nucleotides. Therefore, it is suggested that targeting metabolic pathways can provide novel therapeutic approaches in tumors exhibiting KRAS-mutations (81).

Another pro-tumorigenic mutation in the tumor suppressor genes tuberous sclerosis complex (TSC)1 or TSC2 was recently reported as being linked to metabolic disorders (82). Importantly, the p62/sequestosome-1 (SQSTM1) protein that accumulates in cells with increased mTORC1 with TSC1 or TSC2 mutations, is involved in the pro-tumorigenic process (83). Thus, Lam et al. showed that depleting p62 induces metabolic alterations, including decreased intracellular glutamine, glutamate and glutathione (GSH). Furthermore, p62 attenuation induced modifications in mitochondrial morphology, reduced mitochondrial membrane polarization, increased mitochondrial ROS, and mitophagy markers. All these findings indicate that p62 can be involved in the metabolic pro-tumoral pathways and thus poses a potential therapy target (82). The starch binding domain-containing protein 1 (Stbd1) is involved in autophagy, being a selective autophagy receptor for glycogen. Importantly, Stbd1 is also localized in mitochondria-associated membranes (MAMs). Silencing this protein induced an increase in the spacing between ER and mitochondria and an altered morphology of the mitochondrial network, resulting in mitochondrial dysfunction (84).

The hypoxic microenvironment of tumors induces alterations in gene and protein expression that can lead to increased therapeutic resistance (85). Thus, in a previous study, when HT-29 cells that express hypoxia receptor carbonic anhydrase IX (CA IX), were subjected to CA IX inhibitors, their proliferative capacity was reduced. Furthermore, in a HT-29 xenograft animal model, tumor growth inhibition was obtained upon the utilization of the CAIX-silenced HT-29 tumor cells (86).

The type 1 alpha regulatory subunit (R1a) of cAMP-dependent protein kinase A (PKA) (PRKAR1A) is an important regulator of the serine-threonine kinase activity catalyzed by the PKA holoenzyme (87). In adrenocortical lesions, the identification of PRKAR1A, PDE11A, PDE8B mutations, and defects of mitochondrial oxidation would lead to various tumors initiation, as shown by Stratakis (88). In human B lymphocytes, PRKAR1A-inactivating mutations induce an increased cell cycle rate and decreased apoptosis, thus inactivating PRKAR1A can influence several molecular pathways that control cell cycle and apoptosis (89).

An important research field linking genomic alterations and protein expression is transcriptomics, domain that is also able to detect pro-tumorigenic metabolic traits (90). Indeed, as discussed by Ong and Ramasamy, sirtuin 1 (SIRT1), a member of the histone deacetylase family, inhibits p53 activity and, hence, it is involved in the main cellular physiology processes, including aging, tumorigenesis and reprogramming. Indeed, in aging, SIRT1 expression is closely related to DNA damage, where aged cells are predisposed to tumorigenesis. Interestingly, the Sirt1-p53 axis has dual action acting both as a tumor suppressor and as a promoter, depending on the SIRT1 localization. These authors conclude that the SIRT1-p53 pathway is a potential regulatory axis for aging and tumorigenesis and a possible target of therapeutic strategies (91).

Camacho et al. have recently shown that RNA binding proteins (RBPs) are also involved in metabolism indicating correlation between transcriptomic traits of metabolism and inflammation in cancer (92). These authors have demonstrated that cold-inducible RNA binding protein (CIRP) binds to the TLR4-MD2 complex in serum, and is a damage-associated molecular pattern (DAMP) (92). Furthermore, while CIRP activates the NF-κB pathway, it also alleviates mRNAs that encode pro-inflammatory cytokines – a process important in certain cancer types. In other pathological conditions, such as wound healing, it decreases inflammation tissue regeneration, suggesting that CIRP can modulate inflammation dependent on the specific tissue context (93).

Other key players in the transcriptomics domain are the long non-coding RNAs (lncRNAs) that have been shown to be involved in several pathologies, including cancer metabolism and inflammation. Under normal conditions, lncRNAs are specific for the resident tissues and are subject to strict regulation (94). However, in many cancer types, the aberrant expression of lncRNAs which controls the transcription of pro-tumorigenic genes would drive metabolic pathways that facilitate cancer initiation and progression. Thus, it is argued that lncRNAs can be potential biomarkers for inflammation and metabolic deregulation in cancer and can thus present potential therapeutic targets (92).

Likewise, microRNA (miRNAs or miRs) are post-transcriptional regulators of oncogene expression (95). Recently, it has been shown that the Ras association domain family member 1 (RASSF1) can be a tumor suppressor controlling cell proliferation and apoptosis (96). Specifically, it was demonstrated that the expression of RASSF1 in several types of cancer is reduced, because of the hypermethylation promoter. In this novel mechanism, RASSF1 expression is regulated by miR-193a-3p binding to RASSF1-3'UTR that suppresses mRNA generation and protein expression (96). Thus, Pruikkonen et al. showed that following epigenetic regulation, specific miRNA alterations can contribute to the loss of Rassf1 in cancer cells, inducing the polyploidy of dividing cells (96).

## Metastasis and its metabolic trait

Tumorigenesis is a complex intermingled process where genetic or epigenetic phenomena interact with the energetic homeostasis, organelle activities (e.g., mitochondrial function) and the cellular overall metabolome. These interacting processes are perpetrated in the context of the inflammation mediated tumor microenvironment that can either facilitate tumor progression or dampen its evolution (97, 98). In addition, the changes of tumor cell metabolism triggered by transcriptional programs alterations in association with the inflammatory milieu can further render tumor cell metastasis (99).

In terms of hindering metastasis at metabolic level, two major therapeutic approaches have been proposed: one focusing on increasing the extracellular pH that would obstruct migration, invasion, and metastasis; and the other decreasing the intracellular pH favoring apoptosis (100). In a recent study, it has been shown that pH dysregulation in solid tumors was related to the Warburg effect and hypoxia through the overexpression of NHE1, H+ pump V-ATPase, CA-9, CA-12, MCT-1, and GLUT-1. The activation of these proton exchangers and the associated acidification of the tumor microenvironment is suggestive of suitable therapeutic targets (101).

Another enzymatic process linked to the enhanced glycolysis in cancer cells is the phosphorylation-induced activation of lactate dehydrogenase A (LDHA). LDHA catalyzes the interconversion of pyruvate and lactate and it has been recently reported that the upstream mediators, HER2 and Src kinases phosphorylate LDHA at tyrosine 10 (102). Importantly, in head and neck cancer, as well as in breast cancer cell lines, the expression of Y10-phosphorylated LDHA was positively associated with cell invasion ability and anoikis resistance. Generating cancer cells deficient in DHA or accomplishing LDHA rescue expression has been shown to result in the attenuated metastasis of these cells in a xenograft tumor model. Importantly, the same authors determined that the level of LDHA phosphorylation at Y10 was directly associated with the progression of metastatic disease in a cohort of breast cancer patient biopsies. That study suggested that LDHA Y10 expression can be utilized as a future therapeutic target and/or a prognostic marker (102).

Recently, in the metastasis complex process, cancer stem cells (CSC) were taken responsible for the initiation and development of a neoplastic tissue, but moreover for the metastasis of tumor and the therapeutic resistance. Current treatments cannot specifically target CSCs and thus they are not successful for the complete eradication of the tumor. Overall CSCs can survive under genotoxic/therapeutic toxicity due to their drug efflux pumps equipment, enhanced DNA damage repair machinery and last, but not least special metabolic traits (103).

There are several metabolic portraits of CSC in various solid tumors (104, 105). Thus, CSCs in liver cancer are characterized by enhanced glycolysis, decreased fatty acid biosynthesis (FAS) and OXPHOS. Glycolysis and OXPHOS levels can be found both either increased or decreased in glioma CSCs, whereas their FAS levels are increased. CSCs in breast cancer have an enhanced glycolysis and FAS levels, but decreased OXPHOS. Last, but not least, CSCs in colorectal cancer are characterized by enhanced glycolysis and decreased OXPHOS. These distinct CSC metabolic phenotypes suggest the importance of a thorough investigation when drugs directed toward metabolic deregulations are searched (105).

In a recent report, it has been shown in various metastatic cell lines that by silencing the expression of the mitochondrial protein VDAC1, human glioblastoma (U-87MG), lung cancer (A549), and triple negative breast cancer (MDA-MB-231) cell lines have had an inhibited proliferation. Moreover, by silencing this protein, stemness was inhibited and hence the capacity to develop metastasis. It is interesting that VDAC1 silencing increased the expression of p53 and decreased the expression of HIF-1a and c-Myc; this silencing practically rewired cancer cell metabolism in various types of cancer (e.g., breast, lung cancer, and glioblastoma) (106).

As metastasis is one of the major processes in cancer, leading to tumor cells spreading to other tissues, there is an increased need to understand metabolic requirements of the metastasizing tumor cell as its specific metabolic pathways that further influence on cell signaling and differentiation. Moreover, knowing that tumors are highly heterogeneous, when searching to improve therapy, different metabolic traits characterize resident populations; therefore, different therapeutical targets should be accounted.

## Therapy highlights for targeting mitochondria in apoptosis induction

As elaborated in the previous sections, mitochondria is involved in tumorigenesis through the following main mechanisms: mitochondrial ROS inducing the accumulation of oncogenic DNA mutations and activation of oncogenic signaling pathways (46), accumulation of particular mitochondrial metabolites [e.g., fumarate, succinate, 2-hydroxyglutarate (2-HG), with pro-tumorigenic action (107)] and functional deficits in mitochondrial permeability transition (MPT) that induce the survival of tumor cells (108, 109).

All these mechanisms that are governed by mitochondria are targeted for apoptosis induction in the tumor cell.

Apoptosis is an intrinsic process that regulates physiological events, such as the removal of damaged/un-needed cells and is obligatory for tissue homeostasis (110). This process is fine-tuned in order to differentiate between normal and abnormal cells, including neoplastic cells. During the apoptotic process, the cells die in a controlled manner, in contrast to necrosis, that is an uncontrolled cellular event. Indeed, there are several types of programmed cell death, e.g., autophagic cell death, necroptosis and pyroptosis; however, the only cell death process dependent on mitochondrial pathways that is also caspase-3, -6 and 7-dependent, is apoptosis (111). Apoptosis is sustained by complex regulatory mechanisms and, in oncology, this is one of the biological pathways that controls the number of tumor cells, and hence, tumor growth. Indeed, neoplastic-transformed cells acquire, during their transformation, numerous deregulated intracellular pathways that disrupt the physiology of an array of cellular organelles. Thus, the mitochondria, endoplasmic reticulum, Golgi apparatus, proteasomes, and lysosomes have been reported to exhibit multiple deregulations in tumor cells and subsequently, to present possible therapeutic targets in oncology (112). In fact, in 2018, the Nomenclature Committee on Cell Death (NCCD) had issued important guidelines that provide the hallmarks of cell death characterized by morphological, biochemical and functional parameters; the overview of the main characteristics of cell death hallmarks are presented in Table 1 and reviewed in several papers (113, 114, 116, 124). In all the presented cell death pathways, the complex system of mitochondria is one of the most important, if not the central organelle.

**Table 1:** Main cellular death pathways and their characteristics.

**Cell death pathway**	**Specific features**	**References**
Intrinsic apoptosis	- Induced by various alterations of the extracellular or intracellular milieu and delineated by mitochondrial outer membrane permeabilization. - The key molecule in intrinsic apoptosis is caspase-3 - Plasma membrane integrity is preserved, whereas metabolic activity is partially preserved - During the evolution of the process, rapid clearance by phagocytes (e.g., macrophages) is observed, a process usually known as *efferocytosis*. - A specific variant of intrinsic apoptosis is *anoikis*, which is initiated by the loss of integrin-dependent attachment to the extracellular matrix.	(110)
Extrinsic apoptosis	- Is initiated by distress signals originating from the extracellular setting and detected by plasma membrane receptors, intracellurarly transmitted by caspase-8 and mainly executed by caspase-3, similar to intrinsic apoptosis. - *Death receptors* activated by appropriate ligand binding and *dependence receptors* activated by a specific expression of their respective ligands are involved in extrinsic apoptosis	(110)
Mitochondrial permeability transition (MPT)-driven necrosis	- It is triggered by severe intracellular microenvironment perturbations, such as oxidative stress and cytosolic Ca^2+^ overload - Displays a necrotic morphotype/phenotype - It mostly relies on cyclophilin D as one of the main regulator of the permeability transition pore complex functioning	(113)
Necroptosis	- Initiated by extracellular/intracellular homeostasis alterations - Is a caspase-independent regulated cell death accomplished by the receptor-interacting protein kinases and mixed lineage kinase domain-like protein	(114)
Ferroptosis	- Viewed sometimes as a form of necroptosis, it is initiated by oxidative deregulations at an intracellular level - Reduced glutathione (GSH)-dependent enzyme glutathione peroxidase 4 exerts a constitutive control in ferroptosis - Process inhibitors: Iron chelators and lipophilic antioxidants	(115)
Pyroptosis	- It is a caspase-1-dependent pro-inflammatory cell death induced by alarmins activation - It is executed via inflammasome setting - It is accomplished through membrane integrity loss resulting in extracellular release of pro-inflammatory cytokines, ROS and other intracellular contents	(116)
Parthanatos	- Initiated by the hyperactivation of a specific component of DNA damage response mechanism, namely poly(ADP-ribose) polymerase 1 - Correlated DNA fragmentation is mainly caused by apoptosis inducing factor mitochondria associated 1 and Macrophage migration inhibitory factor - It occurs as a consequence of severe alkylating DNA injury, and as a response to oxidative stress, hypoxia or inflammatory signals	(117)
Entotic cell death	- Type IV cell death or cellular cannibalism - Entosis is defined by a *cell-in-cell* structure in which viable cells engulf other cells - Engulfed cells are subjected to lysosomal degradation by host cells. - Often tumor cells are eliminated by entosis suggesting a tumor suppressive role	(118)
NETotic cell death	- Initially described in neutrophils where neutrophil extracellular traps (NETs) performed NETosis for trapping and degrading various microbes - Now considered as a type of cell death restricted to cell of hematopoietic origin, with ROS involvement	(119)
Lysosome-dependent cell death	- Lysosome membrane permeabilization is a key event - Accelerated by cathepsins, while caspases and mitochondrial outer membrane permeabilization could be an optional event	(120)
Mitotic death	- Merely a variant of intrinsic apoptosis - Led by mitotic catastrophe which is viewed as a control mechanism for mitosis-incompetent cells	(121)
Autophagy-dependent cell death	Type of cell death strongly relying on autophagic machinery	(122)
Immunogenic cell death	Lethal process that activates an adaptive immune response in immunocompetent hosts	(123)

There is an array of compounds, known as mitocans, which target various mitochondrial components in the attempt to induce apoptotic fluxes (125, 126). Therefore, hexokinase inhibitors target hexokinase that is expressed at higher levels in cancer cells, binding both ATP and glucose and resulting in the production of glucose-6-phosphate (G6P), a substrate for the main metabolic pathways. Another class of mitocans, BH3 mimetics are compounds that mimic the Bcl-2 homology-3 (BH3) domains, integral parts of Bcl-2 family of proteins. BH3-only proteins, important players of the mitochondrial-related pathway, can stimulate directly pro-apoptotic Bax-like proteins or can interfere with antiapoptotic Bcl-2 proteins (127). Indeed, Pokrzywinski et al. showed in 2016 that mitochondria-targeted agents (MTA) [e.g., mitoquinone—MitoQ, triphenylphosphonium (TPP+) conjugated agents], can kill breast and lung cancer cells by enhancing ROS production. It was demonstrated that MTAs decrease mitochondrial DNA (mtDNA) integrity in MDA-MB-231 and H23 tumor cell lines, the destruction of these cancer cell lines being based on hindering mitochondrial homeostasis (128). Thiol redox inhibitors, agents targeting voltage-dependent anionic channel (VDAC), and adenine nucleotide translocase (ANT) represent another group of mitocans (129). Importantly, cancer cells have high intrinsic levels of ROS; therefore, compounds that shunt the anti-oxidative potential, such as compounds that oxidize the thiol group or deplete the mitochondrial GSH will increase the ROS-mediated apoptosis of the cell (130).

**Figure 1:**
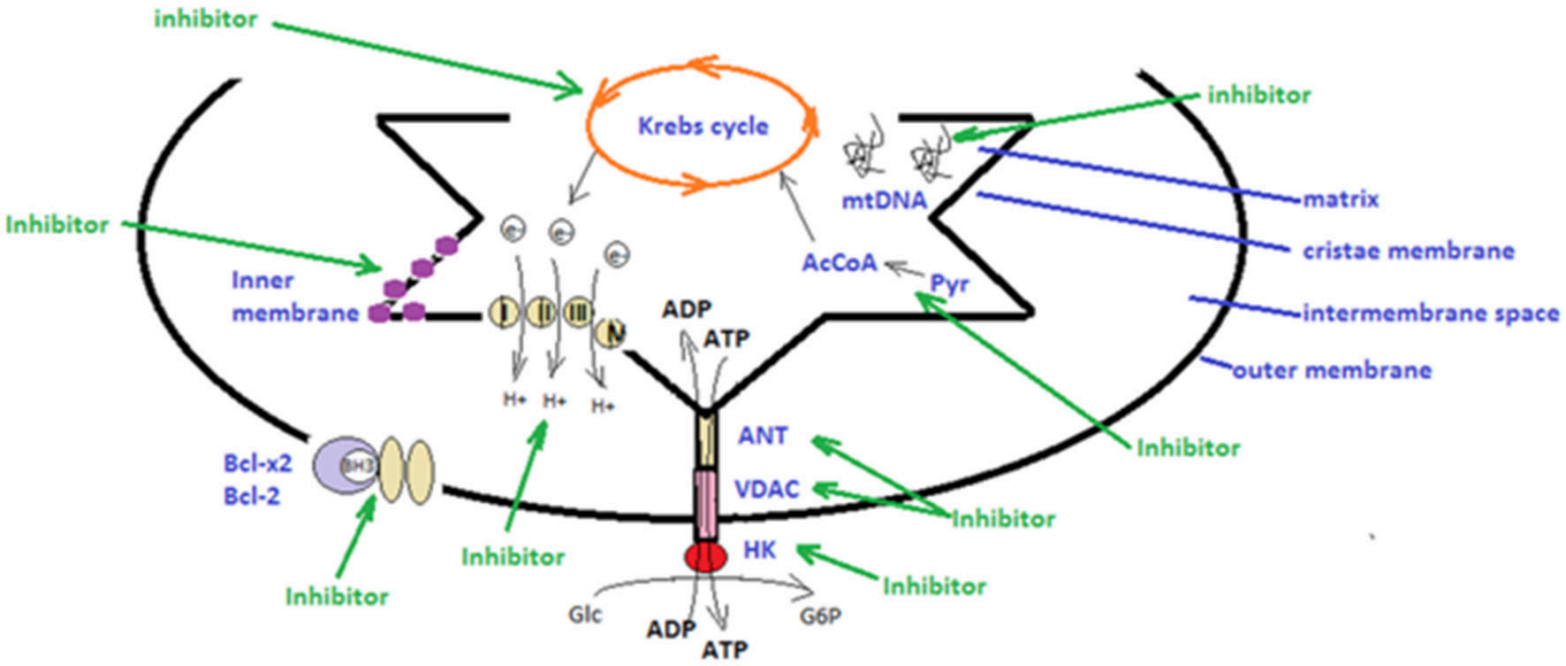
Schematic presentation of strategies utilized in mitochondrial therapeutic targeting. There are several types of mitocans that can target components appending to the outer mitochondrial membrane, to the inter-membrane space, to the *cristae* membrane and to the matrix. Hexokinase (HK), voltage-dependent anionic channel (VDAC) and adenine nucleotide translocase (ANT) can be targeted by inhibitors that will disrupt main metabolic pathways and the anti-oxidative potential of tumor cells (48, 106, 129); The mitochondrial electron transport chain, producing energy by transporting electrons can be targeted by specific inhibitors (131, 132); BH3 mimetics can impair the function of the anti-apoptotic Bcl-2 and Bcl-x2 family proteins (76, 127); lipophilic cations can directly target the mitochondrial inner membrane and hinder mitochondrial transmembrane potential; compounds that affect directly the tricarboxylic acid cycle (Krebs cycle), affect the electrons stream into the electron transport chain (133); inhibitors that target the conversion of pyruvate (Pyr) to acetyl-CoA (AcCoA) hinder the tricarboxylic acid cycle (67); inhibitors that interfere with the mitochondrial DNA (mtDNA) affecting DNA polymerases or transcript levels (46, 83) (green arrows).

Another class of mitocans comprises compounds that affect the mitochondrial ETC (131) and tamoxifen in breast cancer cells will induce apoptosis by hindering FAD-binding site in ETC (132). Importantly, lipophilic cations can directly target the mitochondrial inner membrane. Cancer cells have a high trans-membrane potential compared to non-transformed cells and thus, this type of cations will preferentially target transformed cells (133). Compounds that directly affect the TCA (Krebs cycle) will also affect the electrons streaming into ETC. An example of this compound type is the one that affects the conversion of pyruvate to acetyl-CoA, during TCA. As shown by Truksa et al. some of these compounds comprise the ones that interfere with mtDNA, some affect DNA polymerases, whereas others suppress the D-loop transcript levels (134). A schematic overview of the main mitocan classes is presented in Figure 1. It is promising that several of these compounds have already received clinical approval for the therapy of certain cancer types, including the combination of metformin and 2-deoxyglucose in prostate cancer (135), while BH3 mimetics was approved for chronic lymphocytic leukemia (136). Finally, as already mentioned, tamoxifen, a known drug in estrogen-positive breast cancer, actually interferes with the mitochondrial complex I, namely by the FAD-binding site (132, 137).

Various small molecule classes have been utilized for the induction of the mitochondrial-dependent apoptosis processes (138). Another viable strategy discussed by Fulda is the combination of apoptosis-inducing factors, such as small molecules that antagonize anti-apoptotic Bcl-2 proteins (BH3 mimetics) with drugs targeting the PI3K/Akt/mTOR signaling cascade (139). Indeed, antisense compounds that inhibit Bcl-2 protein production combined with antibodies that bind to death receptors (tumor necrosis factor-related apoptosis-inducing ligand), to MHC (HLA-DR), initiate cancer cell apoptosis. Many types of neoplasias evade pro-apoptotic signals, increasing anti-apoptotic proteins BCL-2, BCL-XL or MCL-1. Therefore, it is concluded that BH3 mimetic drugs can drive the mitochondrial apoptotic pathway in cancer cells; however, given that most cancers can evade apoptotic pathways by different methods, these drugs should be associated with other apoptosis-inducing pathways, such as the PI3K/Akt/mTOR signaling cascade (139). Importantly, BH3 mimetics are gaining increased interest, being currently assessed in clinical trials (140).

The inhibition of the expression of SRC-3 or PFKFB4 in animal models of breast cancer has been shown to lead to reduced tumor growth and metastasis. In breast cancer patients, PFKFB4 expression is increased along with enhanced levels of phosphorylated SRC-3 mainly in estrogen receptor-positive tumors and is associated with a poor survival. Therefore, the results of that study couple the enzyme PFKFB4, an effector of the Warburg pathway, with tumor aggressiveness (41).

The reduction of ATGL enhances oxidative stress and proinflammatory cytokine synthesis metabolically designing an inflammatory tissue microenvironment (29). In this milieu, fibroblasts, endothelial cells and leucocytes are prone to uncontrolled proliferation, further increasing the redox pattern that contributes to tumorigenesis, as proven in medulloblastoma cell lines (30). In hematological malignancies, it has been shown that shingolipid metabolism can be a future therapy target. Thus, reduced cellular ceramide level correlates with tumorigenesis and drug resistance. Drugs that can regulate sphingolipid metabolism can have anti-cancer potency, augmenting the efficacy of additional anti-cancer therapeutics (141).

ROS-dependent therapies that target mitochondria, range from photodynamic therapy, to compounds such as retinoic acid and arsenic trioxide that directly target sub-structures (142). It has been recently shown that ROS can modulate microtubule dynamics in the course of the EB1 phosphorylation process (143).

MPT, a polyprotein complex, regulates mitochondrial homeostasis and apoptosis-related processes, among which is the translocation of the pro-apoptotic proteins Bax/Bcl-2 into the cytosol (144). Therefore, as discussed by Deniaud et al. the permeability transition pore can be an interesting therapeutic target in cancer, as peptides aimed to this complex can induce targeted apoptosis (145). Likewise, Constance and Lim have argued that the disruption of protein-protein interactions with therapy peptides can initiate mitochondrion apoptosis (146). Furthermore, it is well-established that drugs which induce DNA damage can stimulate caspase-independent mitochondrial biogenesis, which further induces both cellular and mitochondrial ROS production. Consequently, these events hinder mitochondrial protein-folding equipment (147). Indeed, Yadav et al. showed that anti-cancer agents that deregulate caspase activation induce the decreased release of mitochondrial cytochrome c in complex-I-deficient cells. Of note, doxorubicin, a DNA-damaging mediator exhibits high affinity to mitochondria, attenuated by complex-I-deficiency but not by complex-II-deficiency. Yadav et al. therefore point out the need to apply subtle strategies upon targeting the mitochondria in cancer therapy (148). Moreover, it has been argued that as pyruvate dehydrogenase kinase (PDK) converts pyruvate to mitochondrial acetyl-CoA, fueling Krebs' cycle, inhibiting this enzyme with, e.g., small interfering RNAs or dichloroacetate (DCA), would shunt glycolysis to glucose oxidation and favor apoptosis (149). Bim, a pro-apoptotic molecule has also been examined as a cancer therapeutic strategy (150). Indeed, Bim favors anoikis, a form of programmed cell death that occurs in anchorage-dependent cells when they detach from the surrounding extracellular matrix. In many tumor cells (e.g., lung cancer, breast cancer, osteosarcoma and melanoma) Bim has been proven to favor anoikis. Tyrosine kinase inhibitors, such as imatinib, gefitinib, or proteasome inhibitors, such as bortezomib, have been investigated as Bim-targeting agents, as recently discussed (151). Moreover, links between mitochondria and other cellular organelles have been scrutinized for anti-cancer therapies. Hence, drugs that hinder microtubule physiology are inducing mitochondrial intrinsic apoptotic pathways. Distinct interconnections between mitochondrial and microtubule intracellular networks would further improve the efficacy of therapeutic drugs (152).

NO, a type of reactive oxygen species (ROS), is an important cellular mediator in both normal cells and neoplastic cells (153). *In vivo*, NO produced by the enzyme NO synthetase (NOS), has been proven to be involved in altered an metabolism, invasiveness, chemoresistance and immune evasion (152, 153). Recently, as discussed by Salimian Rizi et al. in the tumor microenvironment, NO plays a dual role. It can mediate immune responses, while it can induce post-translational protein modifications and tumor-promoting epigenetic modifications. Salimian Rizi et al. suggest that NO metabolism is a potential therapy target in cancer along with other metabolic pathways (154). Likewise, the expression/activity of other ROS-related enzymes, including the antioxidant enzyme catalase can be altered in tumors and, as cancer cells have a high ROS production, this antioxidant enzyme should be thoroughly investigated. Indeed, Glorieux and Calderon discuss that the redox status of tumors and the therapeutic control of catalase expression can aid standard therapy (155).

A recent development in the field is the involvement of autophagy in the process of tumorigenesis (156). In this process, the mitochondria is highly involved, autophagy supplying substrates for mitochondrial metabolic function. The inhibition of TCA alters the production of metabolites that can further deregulate dioxygenases, inducing epigenetic alterations driving tumorigenesis. By contrast, the inhibition of mitochondrial respiratory function and ETC can be injurious to tumorigenesis (157).

A novel mitochondrial inhibitor entered phase I clinical study in patients with pancreatic cancer. CPI-613 is an analog that hinders two mitochondrial enzymes: pyruvate dehydrogenase and α-ketoglutarate dehydrogenase (158, 159). This new mitochondria-related drug was given in combination with modified FOLFIRINOX (160). The disease control, response and complete response rates were improved in comparison to FOLFIRINOX (161). As the results were positive in 2019, continuation of testing in phase II and III are expected (55).

As apoptosis is such an important process in oncology, apoptotic inducers constitute one of the major players in anti-tumoral therapeutical approaches. The development of multi-cellular tumors proves the acquired apoptosis resistance of the tumor cell. The therapeutical armentarium inducing apoptosis implies the use of small molecules, antisense oligonucleotides, monoclonal antibodies, recombinant proteins and various chemical compounds (162) to overcome the adaptability of tumor cells within the established tumor tissue.

Probably the main problem when targeting mitochondria is the modality to differentiate tumor cells in the context where these cells are in close contact with anti-tumor immune cells, in particular CD8+ cytotoxic T lymphocytes. Refined therapeutic approaches where tumor cell are reprogrammed to enter cell growth arrest while immune cells are at least rendered with insensitiveness would have a serious therapeutic potential for cancer therapy (163).

## Summary

**Figure 2:**
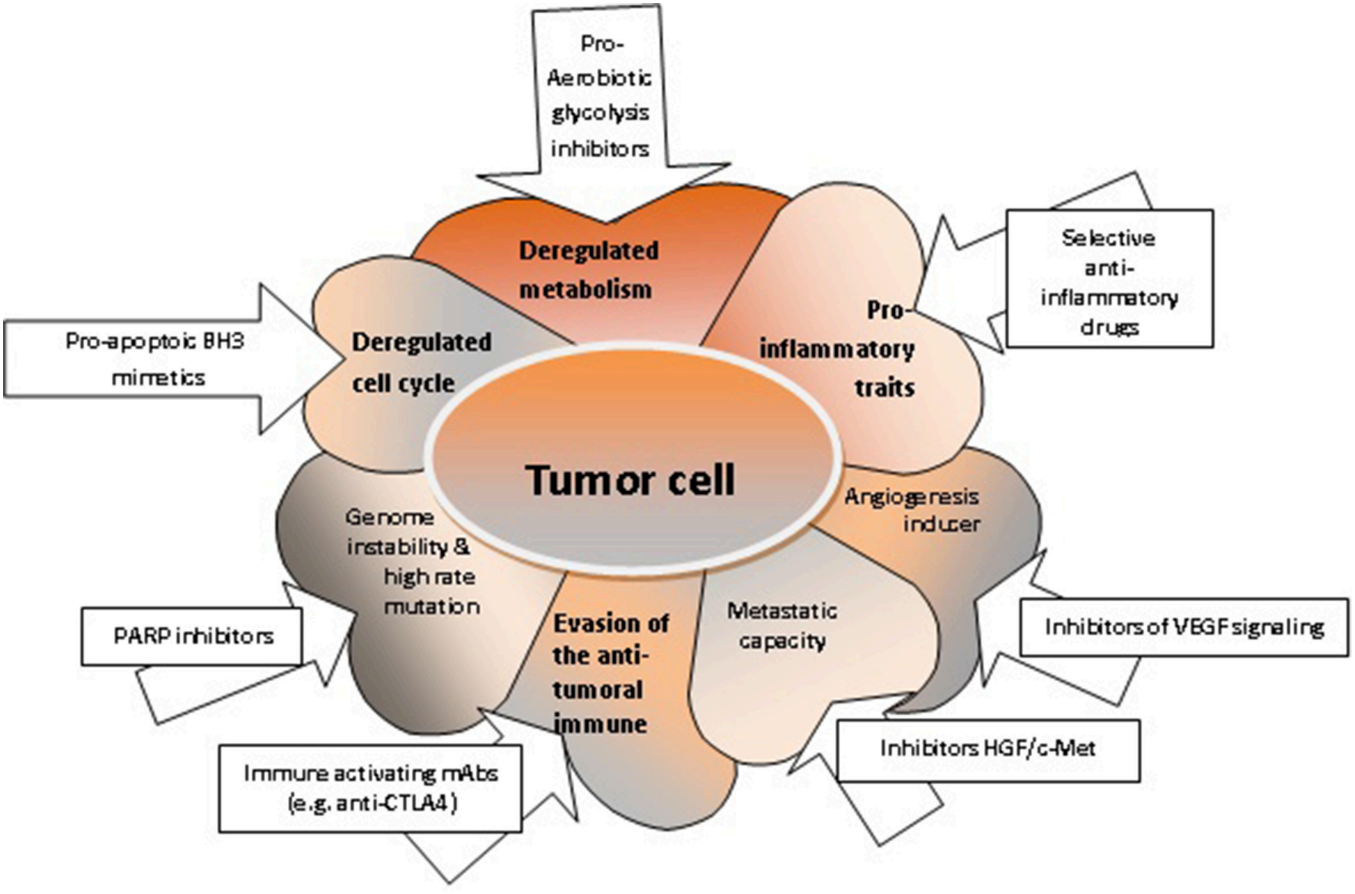
The characteristics of tumor cells and respective targeted therapies. Deregulated metabolism can be targeted with pro-aerobiotic glycolysis inhibitors (52); deregulated cell cycle and increased uncontrolled cellular proliferation can be targeted with pro-apoptotic BH3 mimetics (76, 127); acquired genome instability can be targeted with PARP inhibitors (164); the evasion mechanisms of tumor cell from the anti-tumoral immune response can be controlled by immune activating mAbs (165); enhanced migration capacity of the tumor cell can be targeted with inhibitors HGF/c-Met (166); the angiogenic capacity can be shunted using inhibitors for VEGF signaling; pro-inflammatory properties of the tumor cell can be hindered by selective anti-inflammatory drugs (167).

Cancer metabolism has been the focus of many researchers over the past years. This has resulted in the introduction of metabolic markers to clinical practice for disease monitoring and for the assessment of the therapeutic response. Importantly, a majority of 80% of tumor cells use intracellular signaling pathways that develop aerobic glycolysis, the so-called Warburg effect. Cancer cells utilize enzymes with recently discovered functions, such as PFKFB4, that introduces phosphate groups to proteins, which may contribute to the tumorigenesis of various solid cancers. Moreover, the changes of metabolism can trigger transcriptional programs alterations associated with the inflammatory milieu, accelerated cellular proliferation and metastasis (41). Indeed, deregulated metabolism affects many tumor cell functions, which may constitute therapeutic targets (Figure 2).

One of the major goals of anti-cancer therapy is the induction of the apoptotic machinery and, as the mitochondrial pathway of apoptosis is the main route in this process, mitochondrion physio/pathology is the core of developing novel anti-cancer drugs. There is a panel of molecules that are structurally and functionally appended to the mitochondria and all these molecules can be targeted in anti-cancer therapies. Importantly, these drugs can multi-functionally target the cancer cells. There are various mitochondrial targets that are developed and some of these have already been tested in clinical trials^1^. These drugs can be used individually or in combination with other therapeutic agents. Thus, the development of novel mitochondrial drug delivery systems, nanostructures or multifunctional chemical compounds for targeted cancer therapy is imminent (168). Jointly, the information gathered over the past decades has strengthened the role of the mitochondria in normal physiology and in pathology, a recognition that is highlighted by the emergence of “Mitochondrial Medicine” (169). Recently emphasized, tumorigenesis *per se* was shown as a mitochondrial disease where metabolically high jacked mitochondria becomes highly dependent on glucose and glutamine and, through mitochondrial therapy targets, new therapeutical avenues can be opened (170).

The mechanisms that link metabolic reprogramming, transcriptional regulation, and pro-inflammatory picture of tumorigenesis remain to be elucidated in order to design multi-targeted therapies in cancer.

## Author Contributions

MN, CC, IP, and DZ: paper outline, data acquisition, analysis and interpretation of data, manuscript drafting, critical revision of the manuscript for important intellectual content. GT and DN: data acquisition, analysis and interpretation of data, manuscript drafting, critical revision of the manuscript for important intellectual content. CF, CAS, DAS, and AMT: analysis and interpretation of data, manuscript drafting, critical revision of the manuscript for important intellectual content.

### Conflict of Interest Statement

The authors declare that the research was conducted in the absence of any commercial or financial relationships that could be construed as a potential conflict of interest.

## Footnotes

^1^https://clinicaltrials.gov/ct2/results/details?recrs=a&intr=mitochondria&age=1
